# Association between HLA alleles and adverse drug reactions. Mechanisms involved

**DOI:** 10.1515/almed-2025-0118

**Published:** 2025-09-05

**Authors:** Ana de Malet Pintos-Fonseca, Laura Riesco-Dávila, Serafín Mirete-Bachiller, Israel Nieto-Gañán

**Affiliations:** Microbiology Department, Complejo Hospitalario de Vigo, Pontevedra, Spain; Immunology Laboratory, Servicio de Análisis Clínicos, Complejo Hospitalario de Vigo, Pontevedra, Spain; Immunology Laboratory, Hospital Universitario de Torrecárdenas, Almería, Spain

**Keywords:** HLA, adverse drug reactions, pharmacogenetics, immunology, drug hypersensitivity, severe adverse skin reactions

## Abstract

**Introduction:**

Adverse drug reactions (ADRs) represent a major public health problem. A significant subset of these reactions, particularly delayed-type hypersensitivity reactions and severe cutaneous adverse reactions (SCARs), show a strong association with specific alleles of the human major histocompatibility complex (HLA). The following review aims to summarize the current evidence on the associations between HLA alleles and ADR, covering both well-established associations and those with emerging evidence. The proposed immunological mechanisms for these adverse reactions are also outlined, including hapten/pro-hapten, pharmacological interaction with immune receptors (p-i) and the altered peptide models.

**Content:**

A systematic literature search was conducted in the PubMed database (MEDLINE) through April 2025, independently by the authors. A consensus was then reached, with a total of 56 articles being used in this review.

**Summary:**

A division by groups of drugs is established (antiretrovirals, aromatic antiepileptics, allopurinol, antibiotics and other drugs), indicating for each of them the HLA involved, the proposed mechanism, ethnicity in which the association was described and the degree of association. A table is included to facilitate consultation of key information.

**Outlook:**

HLA alleles play a critical role in the pathogenesis of numerous ADRs, and further studies are needed to clarify HLA-ADRs associations and the mechanisms involved.

## Introduction

Adverse drug reactions (ADRs) are a major cause of morbidity and mortality worldwide, representing a significant burden on healthcare systems [1]. A significant portion of ADRs are unpredictable, not dose-related, and often mediated by immunological mechanisms [2], being drug hypersensitivity reactions (DHRs) particularly relevant. DHRs can range from mild skin rashes to potentially life-threatening systemic reactions, such as severe cutaneous adverse reactions (SCARs), which include Stevens-Johnson syndrome (SJS), toxic epidermal necrolysis (NET), and drug reaction with eosinophilia and systemic symptoms (DRESS), also known as drug hypersensitivity syndrome [3].

In recent decades, research has revealed a strong genetic association between specific human leukocyte antigen (HLA) complex alleles and susceptibility to developing certain DHRs, especially delayed-type reactions (Type IV) [4]. It is well known that the HLA system plays a central role in antigen presentation to T cells, being fundamental for the adaptive immune response [3], [4], [5]. The allelic diversity of the HLA system is enormous (the most polymorphic genetic system in humans), and it has been shown that certain HLA alleles have a particular affinity for certain drugs or their metabolites, triggering an aberrant immune response in carrier individuals [5].

The discovery of these HLA-ADR associations has opened the door to pharmacogenetics in the field of clinical immunology. The identification of genetic biomarkers such as HLA alleles allows patients to be stratified according to their risk of developing specific ADRs before starting drug treatment. This has the potential to personalize therapy, improve patient safety, and reduce the costs associated with the management of ADRs [6].

This review aims to collect and analyze current scientific evidence on the association between HLA alleles and ADRs through an exhaustive search in PubMed. Both clearly established associations replicated in various populations and those with emerging or less conclusive evidence will be addressed. The proposed immunopathogenic mechanisms explaining these associations are briefly summarized and they will be classified by drug groups, also considering other key aspects such as the mechanism, race, and strength of the association in addition to the associated HLA allele. A summary table is included for quick consultation of the most relevant information.

## Methods

A systematic literature search was conducted in the PubMed (MEDLINE) database up to April 2025. Combinations of MeSH (Medical Subject Headings) search terms and free text keywords were used, including: “HLA Antigens”, “Drug Hypersensitivity”, “Adverse Drug Reaction Reporting Systems”, “Pharmacogenetics”, “Stevens-Johnson Syndrome”, “Toxic Epidermal Necrolysis”, “Drug Reaction with Eosinophilia and Systemic Symptoms Syndrome”, and specific drug names (e.g., “Abacavir”, “Carbamazepine”, “Allopurinol”, “Nevirapine”, “Flucloxacillin”, etc.).

Original studies (case-control, cohort), meta-analyses, and systematic reviews investigating the association between specific HLA alleles and clinically well-defined ADRs were included. Studies with high-resolution HLA genotyping were prioritized. Single case reports and letters to the editor without original data were excluded.

The authors independently evaluated the titles and abstracts of the retrieved articles to determine their eligibility, excluding those where consensus was not reached. Of a total of 98 different articles obtained from the search conducted by each author, 56 articles were finally selected for this review. Relevant information was extracted from the selected articles, including: implicated drug, type of ADR, associated HLA allele(s), studied race, allele frequency in case-control studies, odds ratio (OR) with 95 % confidence interval (CI), and proposed immunological mechanisms. Where was possible, the strength of the association was classified according to the OR value (considering strong if OR>10, moderate if 2≤OR≤10, and weak if OR≤2).

## Immunological mechanisms associated with HLA-mediated ADRs

**Figure 1:**
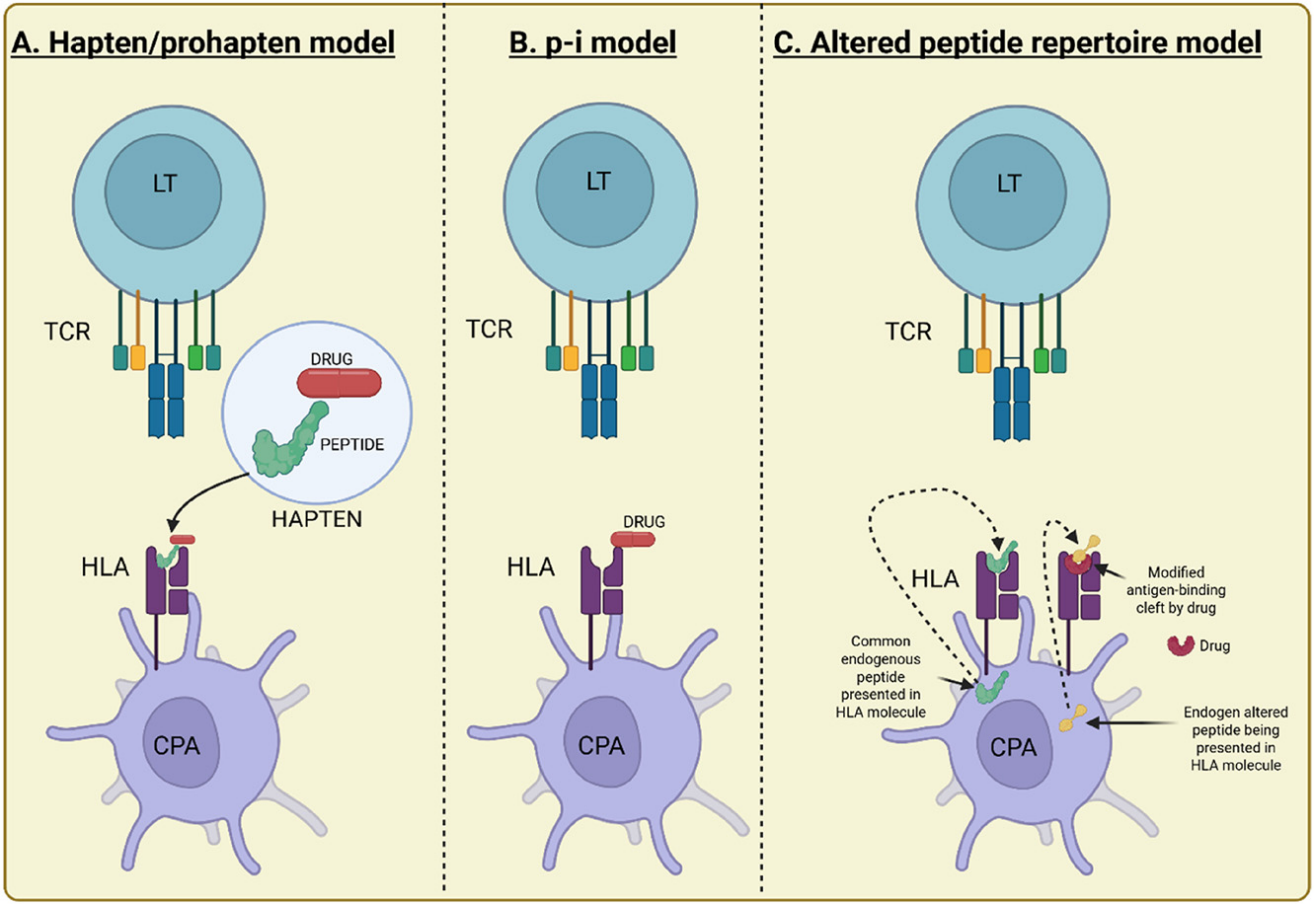
Main proposed mechanisms for HLA-mediated ADRs. (A) Hapten/pro-hapten model: The drug (or its metabolite) binds covalently to a cellular protein, is processed, and presented as a modified peptide or hapten by the HLA molecule to a specific T cell. (B) p-i model: The drug interacts directly and non-covalently with the HLA-peptide complex or the TCR, causing T cell activation without the need for processing. (C) Altered peptide model: The drug binds non-covalently to the HLA molecule, altering the repertoire of endogenous peptides presented. The T cell recognizes this new altered HLA-peptide complex as foreign, causing an immune response. APC: antigen-presenting cell; HLA: human leukocyte antigen; TCR: T cell receptor. Created in https://BioRender.com.

The presentation of drugs or their metabolites by specific HLA molecules to T cells is the central event in many immunologically mediated DHRs. Several models (Figure 1) have been proposed to explain how drugs can activate T cells in an HLA-restricted manner [5], 7], 8]:

### Hapten/pro-hapten model

This is the classical model. Chemically reactive drugs (haptens) or their metabolites (pro-haptens that become reactive after metabolism) bind covalently to endogenous proteins (carriers). These drug-protein conjugates are processed by antigen-presenting cells (APCs) and presented as modified peptides in the peptide-binding groove of HLA molecules. Specific T cells recognize these modified peptide-HLA complexes, triggering the immune response [9]. While this mechanism might be relevant for some reactions (e.g., penicillin allergy), it doesn’t fully explain the high specificity observed for many HLA-associated ADRs [5], 7].

### Pharmacological interaction with immune receptors model (p-i model)

This model proposes that some drugs can bind directly and non-covalently to HLA molecules or the T-cell receptor (TCR) without the need for metabolic processing or covalent binding to peptides [10]. This interaction can occur in several ways:

– **Binding to the HLA** **peptide-binding groove:** The drug might bind within the groove normally occupied by peptides, either altering the repertoire of self-peptides presented or creating a novel antigenic surface recognized by T cells [11], 12]. This is thought to be a key mechanism for the abacavir-HLA-B*57:01 association [13]*. *The drug binds within the F-pocket of the HLA-B*57:01 binding groove, altering its conformation and the set of self-peptides it can bind and present, leading to the activation of specific Vβ T-cell subsets [13].
– **Binding outside the** **peptide-binding groove:** The drug could bind elsewhere on the HLA molecule, potentially altering its interaction with the TCR or co-receptors [7].
– **Direct TCR interaction:** The drug might interact directly with the TCR, potentially stabilizing a low-affinity interaction with a self-peptide-HLA complex or triggering signaling in the absence of HLA binding [5].

### Altered peptide model

Similar to the p-i model, this model suggests that the drug binds non-covalently to the HLA molecule, but instead of directly stimulating T cells, it alters the repertoire of endogenous peptides that can bind to and be presented by that specific HLA molecule. T cells would then recognize these new HLA-peptide complexes (with endogenous peptides that would not normally be presented or would be presented with low affinity) as foreign, initiating the immune response [13]. This model has been proposed to explain the association between the HLA-B*58:01 allele and allopurinol ADRs [14].

The common outcome of these interactions is the activation of drug-specific T cells (often CD8+ T cells for class I associations like HLA-B and CD4+ T cells for class II associations like HLA-DR/DQ). Activated T cells proliferate and release cytotoxic mediators (e.g., perforin, granzymes) and pro-inflammatory cytokines (e.g., IFN-γ, TNF-α), causing tissue damage characteristic of the specific ADR phenotype. The specific HLA allele dictates the binding affinity and presentation of the drug or drug-peptide complex, explaining the high specificity of these associations [13].

## HLA-ADRs associations

The most relevant associations between HLA alleles and ADRs are described below, classified by drug groups, including the most relevant information for each of them. Table 1 summarizes the most relevant aspects of each drug.

**Table 1:** Main associations between HLA alleles and adverse drug reactions.

Drug	HLA allele	Main ADR	Proposed mechanism	Ethnicity	Association strength (OR)	References
Abacavir	HLA-B*57:01	AHS	p-i/Altered peptide	Caucasians, Global	>100	[11], 15], 17]
Nevirapine	HLA-C*08	MPE, SJS/TEN	Unknow	Asians (Thai)	∼10–20	[18]
	HLA-DRB1*01:01	Hepatotoxicity	Unknow	Caucasians	Moderate	[19]
	HLA-B*14:02	Hepatotoxicity	Unknow	Africans	Weak	[20]
	HLA-C*04	SJS/TEN	Unknow	Japanese	Weak	[21]
Carbamazepine	HLA-B*15:02	SJS/TEN	p-i	Southeast Asian	>100	[12], 22], 30]
	HLA-A*31:01	MPE, DRESS, SJS/TEN	Unknow (p-i?)	Europeans, Japanese, Koreans	5–15	[23], 24], 27]
Oxcarbazepine	HLA-B*15:02	SJS/TEN	p-i (similar to CBZ)	Southeast Asian	Strong (>50)	[30], 31]
Phenytoin	HLA-B*15:02	SJS/TEN	p-i (similar to CBZ?)	Southeast Asian	5–10	[32]
	HLA-B*13:01	DRESS	Unknow	Asians	Moderate-strong	[33]
Lamotrigine	HLA-B*15:02	SJS/TEN, MPE	Unknow (p-i?)	Southeast Asian	Moderate-weak	[35]
	HLA-B*24:02	SJS/TEN, MPE	Unkmow (p-i?)	Koreans	Weak	[36]
	HLA-B*38	SJS/TEN, MPE	Unknow (p-i?)	Europeans	Weak	[36]
	HLA-A*33:03	SJS/TEN, MPE	Unknow (p-i?)	Indians	Weak	[36]
	HLA-B*58:01	SJS/TEN, MPE	Unknow (p-i?)	Asians	Weak	[37]
Allopurinol	HLA-B*58:01	SCARs (SJS/TEN, DRESS)	Altered peptide/p-i	Asians (Han, Thai, Kor)	>100	[14], 38], 39]
Flucloxacillin	HLA-B*57:01	DILI (Cholestatic/Mixed)	Hapten/p-i?	Europeans	∼80	[43], 44]
Amoxicillin-Clav.	HLA-DRB1*15:01/DQB1*06:02	DILI (Cholestatic/Mixed)	Unknow	Europeans	2–5	[45]
Sulfamethoxazole	HLA-B*13:01	DRESS	Hapten/p-i?	Asians	Weak to moderate	[46]
	HLA-B*38:02	SJS/TEN, MPE	Hapten/p-i?	Various	Weak to moderate	[46]
	HLA-B*15:02	SCARs	Hapten/p-i?	Various	Weak to moderate	[47]
Vancomycin	HLA-A*32:01	DRESS	Unknow	European	Weak to moderate	[48]
Dapsone	HLA-B*13:01	Hipersensibility (DHS/DRESS)	Unknow	Asians (Chinese, Thai)	>20	[49], 50]
Methimazole/PTU	HLA-B*38:02	Agranulocytosis	Unknow	Caucasians	10–20	[51]
Lapatinib	HLA-DRB1*07:01/DQA1*02:01	Hepatotoxicity	Unknow	Various	∼5–15 (moderate-strong)	[52], 53]
NSAIDs	HLA-B*15:02	SJS/TEN	Unknow	Chinese	Weak	[54]
	HLA-DPB1*03:01	NERD	Unknow	Various	Weak	[54]
	HLA-A*29, HLA-B*44	SJS/TEN	Unknow	Europeans	Weak	[55]

DRESS, drug reaction with eosinophilia and systemic symptoms; AHS, abacavir hypersensitivity syndrome; CBZ, carbamazepine; DILI, drug-induced liver injury; MPE, maculopapular eruption; NSAIDs, non-steroidal anti-inflammatory drugs; p-i, pharmacological interaction with immune receptors; PTU, propylthiouracil; ORs, are approximate and may vary significantly between studies and populations; SCARs, severe cutaneous adverse reactions; SJS, Stevens-Johnson syndrome; TEN, toxic epidermal necrolysis.

### Antiretrovirals

#### Abacavir

The HLA-B*57:01 allele is linked to abacavir hypersensitivity syndrome (AHS), a potentially fatal multi-organ reaction occurring in 5–8% of patients, typically within the first six weeks of therapy [15]. This represents one of the strongest known HLA-ADR associations [6]. The proposed mechanism is the p-i (pharmacological interaction with immune receptors) model, where abacavir binds non-covalently to the peptide-binding groove of HLA-B*57:01, altering its specificity. This change allows the binding of new endogenous peptides, which in turn leads to the expansion of specific cytotoxic CD8+ T cells [11], 16]. This strong association is observed in diverse populations, particularly Caucasians, and the prevalence of the HLA-B*57:01 allele varies, being higher in some European groups and lower in Asian and African populations [15]. The degree of association is very strong, with an odds ratio (OR) often exceeding 100. The HLA-B*57:01 test has a negative predictive value near 100 % and a positive predictive value around 50–60 % [17], making pre-treatment screening a standard practice that has virtually eliminated AHS in clinical settings [6].

#### Nevirapine

This non-nucleoside reverse transcriptase inhibitor is associated with hypersensitivity reactions (including SJS/TEN and DRESS) and hepatotoxicity, linked to several HLAs. These include HLA-C*08, HLA-B*35:05 linked to skin reactions in Thais and other Asian populations [18], HLA-DRB1*01:01 to hepatotoxicity in Caucasians [19], additional associations for hepatotoxicity with HLA-B*14:02 in African populations [20] and HLA-C*04 for SJS/TEN in Japanese individuals [21]. The proposed mechanism is not fully elucidated but is possibly related to the p-i or altered peptide models, with the diversity of associated alleles suggesting different mechanisms may be at play depending on the specific adverse drug reaction or population [18], 19]. The association is moderate to strong, with variable ORs (e.g., OR ∼10–20 for HLA-C*08 in Thais with a skin rash) [18], but screening is not routinely performed due to the lower strength compared to abacavir and the involvement of multiple alleles.

### Aromatic antiepileptics

#### Carbamazepine

Carbamazepine is linked to several HLA alleles, most notably HLA-B*15:02, which is strongly associated with Stevens-Johnson syndrome (SJS) and toxic epidermal necrolysis (TEN) [22]. Other associated alleles include HLA-A*31:01, linked to a broader spectrum of ADRs like DRESS, SJS/TEN, and maculopapular exanthema (MPE) [23], 24], and HLA-B*15:11 (and other B75 group alleles) associated with SJS/TEN in some Asian populations [25]. The proposed mechanism for HLA-B*15:02 involves the p-i model, where carbamazepine interacts directly with the HLA-B*15:02-peptide complex and the T-cell receptor (TCR) to activate CD8+ T cells [12], 26]. The mechanism for HLA-A*31:01 is less clear but may involve the p-i or altered peptide model with the activation of CD8+ and/or CD4+ T cells [27]. These associations are highly ethnicity-specific: the HLA-B*15:02 link is almost exclusive to Southeast Asian populations (Han Chinese, Thai, Malaysian, Indian) and is rare in Caucasians and Japanese [22], 28], whereas the HLA-A*31:01 association is found in European, Japanese, and Korean populations [23], 24], 29], and the HLA-B*15:11 association is found in Thais [25]. The degree of association for HLA-B*15:02 with SJS/TEN in Asians is very strong (OR>100) [21], leading to recommended screening in these populations [30]. For HLA-A*31:01, the association is strong in Thai population [25].

#### Oxcarbazepine

Oxcarbazepine is primarily associated with the HLA-B*15:02 allele and is known to cause SJS/TEN, demonstrating cross-reactivity with carbamazepine [31]. The proposed mechanism is believed to be similar to that of carbamazepine, likely following the p-i model. This association is concentrated in Southeast Asian populations where the allele is prevalent [31]. The degree of association is strong, with an odds ratio for SJS/TEN similar to that of carbamazepine, and as a result, clinical guidelines that recommend screening for carbamazepine typically extend this recommendation to oxcarbazepine [30].

#### Phenytoin

Phenytoin is associated with ADRs like SJS/TEN and DRESS, with links to several HLA alleles depending on ethnicity. The associated HLAs include HLA-B*15:02 in Southeast Asia [32], HLA-B*13:01 (associated with DRESS) in some Asian populations [33], and HLA-B*56:02/04 (associated with SCARs) in Thai populations [34]. The proposed mechanism is likely the p-i model or an altered peptide model. The degree of association is strong for HLA-B*15:02 in Asians (OR ∼5–10) [32] and moderate to strong for the other alleles within their corresponding populations [33], 34].

#### Lamotrigine

Lamotrigine is mainly associated with SJS/TEN and MPE, though its HLA associations are less consistent than those for carbamazepine. Associated alleles include HLA-B*15:02 (with a weak to moderate link) in Southeast Asia [34], HLA-A*33:03 in Indians [35], 36], HLA-B*58:01 in specific Asian populations [37], HLA-A*24:02 in Koreans [36] and HLA-B*38 in Europeans [35]. The mechanism is not well defined but may involve the p-i model. Overall, the degree of association is generally considered weak to moderate compared to carbamazepine, and the clinical utility of performing HLA screening before starting lamotrigine remains unclear [35], 36].

#### Allopurinol

Allopurinol is strongly associated with the HLA-B*58:01 allele, and it is one of the most common drug causes of severe cutaneous adverse reactions (SCARs), such as SJS/TEN and DRESS, worldwide [38], 39]. The proposed mechanism is the altered peptide model, where allopurinol’s active metabolite, oxypurinol, is thought to bind non-covalently to the HLA-B*58:01 molecule. This binding modifies the repertoire of presented peptides, leading to the activation of specific drug-responsive CD8+ T cells [14], 40]. This very strong association is most prominent in Asian populations like Han Chinese, Thais, and Koreans, where the allele frequency can be as high as 15–20 % [38], 39], but it is also observed in European and African populations where the allele is less common [41]. The degree of association is very strong, with an odds ratio greater than 100 in many populations, which has led to clinical recommendations for HLA-B*58:01 screening in high-risk populations before initiating treatment [42].

### Antibiotics

#### Flucloxacillin

Flucloxacillin is associated with the HLA-B*57:01 allele in cases of drug-induced liver injury (DILI), specifically of the cholestatic or mixed type [43]. The mechanism is not completely established but could involve either the presentation of reactive metabolites via a hapten/pro-hapten model or a direct interaction with HLA-B*57:01 through the p-i model, leading to an immune response against the liver [44]. This association has been mainly described in European populations (UK, Scandinavia, Germany), where HLA-B*57:01 has a significant prevalence [43]. The degree of association is very strong, with an odds ratio of approximately 80; however, routine screening is not widely implemented due to the relatively low incidence of flucloxacillin-induced DILI and considerations of cost-effectiveness [43].

#### Amoxicillin-clavulanate

Amoxicillin-clavulanate is associated with drug-induced liver injury (DILI), predominantly of the cholestatic or mixed type. The associated HLAs are HLA-DRB1*15:01 and HLA-DQB1*06:02, two alleles that exhibit high linkage disequilibrium, and some studies have also reported a link with HLA-A*02:01 [45]. The proposed mechanism is unknown, but the association with class II HLA alleles suggests an important role for CD4+ T cells. This association has been described mainly in populations of European descent and is considered moderate, with an odds ratio of approximately 2–5 [45].

#### Sulfamethoxazole (component of cotrimoxazole)

Sulfamethoxazole is linked to a broad spectrum of ADRs, including MPE, SJS/TEN, DRESS, and cytopenias. Its HLA associations are generally weaker and more specific to the ADR and population, with several alleles implicated. For example, HLA-B*13:01 is associated with DRESS and HLA-B*38:02 with SJS/TEN in some Asian populations [46], while HLA-B*15:02 has also been linked to SCARs [47]. The proposed mechanism likely involves reactive metabolites like nitrososulfamethoxazole acting as haptens, although the p-i model may also be involved [9]. The ethnicity-based associations are variable, and the overall degree of association is generally weak to moderate [46].

#### Vancomycin

Vancomycin infusion reaction (previously “Red Man Syndrome”) is primarily rate-related, but DRESS can occur. An association between vancomycin-induced DRESS and HLA-A*32:01 has been reported in European ancestry populations [48].

### Other drugs

#### Dapsone

Dapsone is associated with the HLA-B*13:01 allele, which is linked to dapsone hypersensitivity syndrome (DHS), a condition similar to DRESS [49], 50]. The mechanism remains unclear but is possibly related to reactive metabolites like hydroxylamine and an altered peptide presentation. This association is found mainly in Asian populations, particularly in China and Thailand [49], 50], and is considered strong, with an odds ratio greater than 20 [49].

#### Anti-thyroid drugs (methimazole, propylthiouracil)

Anti-thyroid drugs such as methimazole and propylthiouracil are associated with the HLA-B*38:02 allele in cases of agranulocytosis [51]. The proposed mechanism is uncertain but may involve an immunological reaction directed against neutrophil precursors. This association has been described in Caucasian populations and is considered to be of moderate to strong significance, with an odds ratio of approximately 10–20 [51].

#### Lapatinib

This tyrosine kinase inhibitor used in cancer therapy can cause hepatotoxicity. HLA-DRB1*07:01 and HLA-DQA1*02:01 have been linked to increased risk [52], 53].

#### Non-steroidal anti-inflammatory drugs (NSAIDs)

Although hypersensitivity reactions to NSAIDs are common, clear HLA associations are not as consistently defined as with other drugs. However, some studies suggest potential links. For instance, oxicam-induced SJS/TEN has been linked to HLA-B*15:02 in Han Chinese populations [54]. Additionally, NSAID-exacerbated respiratory disease (NERD) has complex genetics, but some studies point towards associations with HLA class II alleles like HLA-DPB1*03:01 [54]. Other proposed associations, such as between specific NSAIDs and cutaneous reactions with alleles like HLA-A*29 and HLA-B12 (B*44), require further validation [55].

## Conclusions and future perspectives

HLA alleles play a critical role in the pathogenesis of numerous idiosyncratic ADRs, primarily through mechanisms involving aberrant T-cell activation triggered by drug-HLA interactions. Knowledge of these associations, especially the strongest ones, has already transformed clinical practice for certain drugs, making it possible to prevent potentially fatal reactions through pharmacogenetic screening for specific HLA alleles.

However, several key issues must be considered regarding HLA-ADR associations. These links are often specific to certain ethnicities, such as the different genetic markers for carbamazepine risk in Asian vs. European populations, which necessitates studies across diverse groups [22], 23]. The predictive strength of these associations also varies, as seen with the HLA-B*57:01 marker for abacavir, which has an excellent negative predictive value but only a 50–60 % positive predictive value, indicating that other factors contribute to the reaction [17]. Furthermore, the precise biological mechanisms behind many HLA-ADR connections are not fully understood, and research to identify new biomarkers is ongoing but often yields weaker associations that require more validation [5], 7], 8]. On the other hand, identifying new HLA biomarkers for other ADRs remains an active area of research. Drugs such as sulfonamides, lamotrigine, or even some immune checkpoint inhibitors used in oncology show signs of association with certain HLA alleles, but often with lower ORs or in specific populations, requiring validation in larger and more diverse cohorts [35], 46]. Furthermore, it should be noted that HLA-ADR associations can be influenced by other genetic and environmental factors [23]. Finally, the clinical implementation of HLA screening faces significant logistical and economic barriers, including test cost and availability, the need for professional education, and integration into electronic health systems [6].

For all this, future research in the field of HLA-ADR associations should take into account some of the following areas: identifying and validating new associations for more drugs in diverse populations; deepening the understanding of the underlying immunological mechanisms at a molecular level [11], 16]; using genome-wide association studies (GWAS) to discover other genetic and environmental risk factors [56]; developing more accurate prediction algorithms that integrate HLA information with other clinical data; and performing cost-effectiveness analyses to evaluate the feasibility of a broader and more equitable clinical implementation of these pharmacogenetic tests.

In summary, HLA pharmacogenetics has proven to be a powerful tool for improving drug safety. Therefore, research in this area must continue to advance towards more personalized and safer medicine.
